# Lymphomatous transformation in mass-forming chronic active EBV-associated enteritis initially misdiagnosed as ulcerative colitis: A case report

**DOI:** 10.1097/MD.0000000000049616

**Published:** 2026-07-10

**Authors:** Tao Yang, Liang Ding, Mengjia Tao, Mengping Chen, Yuehua Qin

**Affiliations:** a Department of Gastroenterology, Shaoxing People’s Hospital, The First Hospital of Shaoxing University, Shaoxing, China; b Department of Pathology, Shaoxing People’s Hospital, The First Hospital of Shaoxing University, Shaoxing, China; c Department of Hematopathology, Shaoxing People’s Hospital, The First Hospital of Shaoxing University, Shaoxing, China.

**Keywords:** biologic therapy, chronic active Epstein–Barr virus disease, gastrointestinal involvement, lymphoma, ulcerative colitis

## Abstract

**Rationale:**

Chronic active Epstein–Barr virus (EBV) disease is a group of refractory and progressive lymphoproliferative disorders characterized by both inflammatory and clonal proliferative features. Gastrointestinal involvement is highly uncommon and can often be misdiagnosed as inflammatory bowel disease.

**Patient concerns:**

A 63-year-old man presented with diarrhea and bloody stools and was initially diagnosed with ulcerative colitis at a local hospital. After multiple courses of biologic therapy, progressive development of a mass-forming lesion was observed on serial endoscopy.

**Diagnoses:**

Detection of EBV nucleic acid in serum and colon biopsies showed a high viral load. Serial pathological consultations confirmed the diagnosis of EBV-associated lymphoproliferative disorder. Positron emission tomography/computed tomography revealed disease progression involving the bone marrow, and bone marrow examination confirmed peripheral T-cell non-Hodgkin lymphoma.

**Interventions:**

Biological therapy was discontinued. Allogeneic hematopoietic stem cell transplantation was recommended, but the patient declined. After progression to lymphoma occurred, chemotherapy was administered.

**Outcomes:**

The patient responded poorly to chemotherapy, with rapid disease progression, and ultimately succumbed to multisystem organ failure.

**Lessons:**

This rare case underscores the critical importance of differentiating between chronic active EBV-associated enteritis and inflammatory bowel disease, as their management strategies and prognoses are divergent. The clinical course of chronic active EBV-associated enteritis is aggressive, and the prognosis is poor. Misdiagnosis may delay treatment and potentially accelerate disease progression.

## 1. Introduction

The Epstein–Barr virus (EBV) is a ubiquitous virus that has infected over 90% of people. EBV typically infects B cells, but can also infect T or NK cells, which relates to poor prognosis. Chronic active EBV disease is a systemic, progressive disease caused by EBV infection of T cells and/or NK cells, characterized by both inflammatory and clonal proliferative features. It is refractory and can lead to various lymphoproliferative disorders (LPD) such as infectious mononucleosis, malignant lymphoma, and hemophagocytic syndrome.^[1]^ On rare occasions, the intestine may also be involved, leading to chronic active EBV-associated enteritis (CAEAE). It commonly presents with ulcers, edema, and erosions under endoscopy, making it clinically difficult to distinguish from inflammatory bowel disease (IBD).^[2]^ Here, we present a mass-forming CAEAE, which we believe to be the first such case and finally developed into lymphoma.

## 2. Case presentation

A 63-year-old man was diagnosed with ulcerative colitis (UC) at a local hospital in December 2021, with diarrhea and bloody stools. The patient had a 10-year history of Hodgkin lymphoma, treated with the doxorubicin, bleomycin, vinblastine, dacarbazine regimen, and follow-up evaluations have shown stable disease. Following the UC diagnosis, 4 doses of vedolizumab were administered, and his gastrointestinal symptoms improved. Colonoscopy reevaluation showed improvement 6 months later. However, colon biopsy pathology suggested LPD. Biological therapy was subsequently suspended, but diarrhea symptoms recurred.

**Figure 1.**
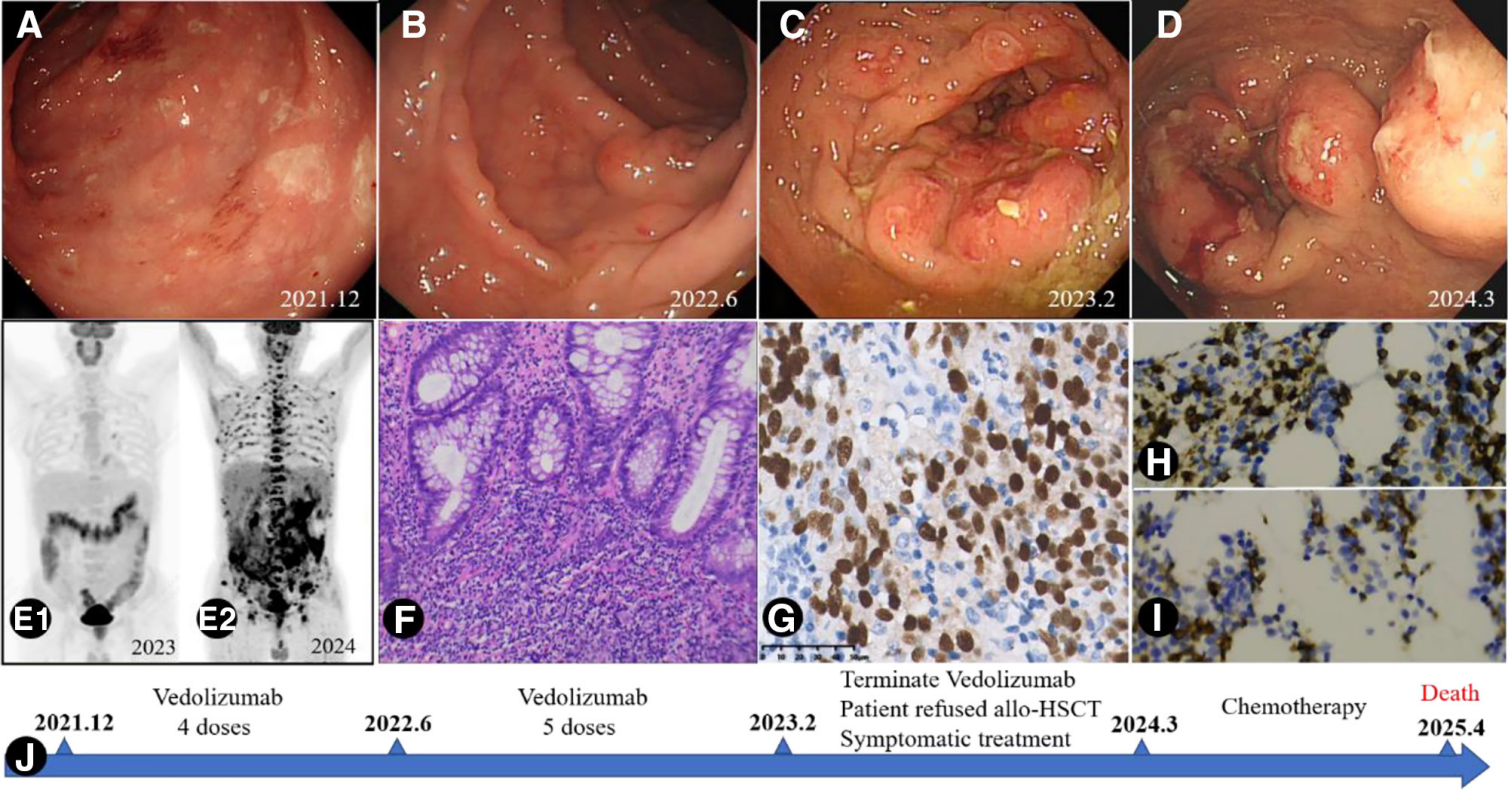
(A, B, C, D) Four serial colonoscopies demonstrated progressive disease evolution with mass-forming transformation. (E1, E2) Two serial consecutive PET/CT scans demonstrated disease progression from colonic confinement to bone marrow involvement. (F) Histopathology of colon biopsies showed chronic active inflammation with marked lymphoid hyperplasia (H&E stain). (G) EBER-ISH of colon biopsies was positive. Immunohistochemical staining for CD3 (H) and CD8 (I) was positive in bone marrow examination (magnification, F in 200×, G, H, I in 400×). (J) The timeline of the patient’s disease occurrence and interventions. CT = computed tomography, EBER-ISH = EBV-encoded RNA in situ hybridization, EBV = Epstein–Barr virus, PET = positron emission tomography.

Subsequently, the patient presented to our hospital 2 months after undergoing colonoscopy. However, due to concealment of the medical history (including the initial diagnostic data and LPD), 5 additional doses of vedolizumab were re-administered, after which the patient’s symptoms improved. Post-treatment laboratory tests showed mild anemia (Hb 118 g/L), elevated C-reactive protein level (16 mg/L), increased erythrocyte sedimentation rate (92 mm/h), positive occult blood stool, normal fecal calprotectin (287.3 μg/g before treatment), negative stool culture, and negative tuberculosis result. An abdominal computed tomography scan found diffuse mural thickening of the rectum with multiple enlarged lymph nodes in the abdominopelvic region. Gastroscopy revealed atrophic gastritis. Colonoscopy demonstrated multiple mucosal elevations with erosions, most prominent in the rectosigmoid region (Fig 1C). The pathologists reported chronic active inflammation with marked lymphoid hyperplasia and no significant crypt architectural distortion (Fig. 1F). EBV-associated enteritis and LPD were suspected rather than UC after a careful review of 3 serial colonoscopies (the initial colonoscopy showed irregular shallow ulcers of varying sizes in the colon, with normal mucosa between the ulcers; Fig. 1A–C). Subsequently, serum EBV-DNA testing revealed a viral load of 7.9 × 10^3^ copies/mL, and EBV-encoded RNA in situ hybridization was positive in colon biopsies from serial colonoscopies, including the biopsy from the initial colonoscopy (Fig. 1G). Serial pathological consultations confirmed the diagnosis of EBV-associated LPD. ^18^F-fluorodeoxyglucose-positron emission tomography/computed tomography showed uptake in the entire colorectum (Fig. 1E1). To exclude lymphoma, we reexamined colonoscopy with multiple-site pathological biopsies, but no evidence of lymphoma was found in any specimen. Biological therapy was discontinued. Bone marrow examination and allogeneic hematopoietic stem cell transplantation (allo-HSCT) were recommended, but the patient declined. In view of the uncertain efficacy and potential side effects of non-HSCT treatments (including antiviral drugs, chemotherapy, immunotherapy, and surgical treatment), the patient also refused these options. Symptomatic treatment was administered along with a recommendation for close monitoring.

However, the patient did not return for follow-up until 1 year later. Colonoscopy demonstrated disease progression (Fig. 1D), but biopsies showed no evidence of lymphoma. The patient subsequently developed recurrent high fever refractory to antibiotics. Repeat positron emission tomography/computed tomography revealed extensive skeletal uptake beyond the already noted colonic involvement, suggestive of hematologic malignancy (Fig. 1E2). Subsequent bone marrow examination confirmed peripheral T-cell non-Hodgkin lymphoma (the lymphoid cells were EBV-encoded RNA positive (EBER+), CD3+, Fig. 1H; CD8+, Fig. 1I), while laboratory findings suggested hemophagocytic lymphohistiocytosis (fever, splenomegaly, cytopenia, hypofibrinogenemia, hemophagocytosis, elevated serum ferritin). The patient received cyclophosphamide, vincristine, doxorubicin, and prednisone chemotherapy but demonstrated poor response with rapid disease progression, ultimately succumbing to multisystem organ failure. The timeline of the patient’s disease occurrence and interventions applied are summarized in Figure 1J.

## 3. Discussion

Chronic active EBV disease is a rare disorder, particularly in the gastrointestinal tract, and usually manifests as LPD rather than simple inflammation. The small intestine and colon are the most frequently involved sites. Endoscopically, lesions predominantly present with ulcers of varying shapes, depths, and sizes.^[2]^ During disease progression, CAEAE may be complicated by hemophagocytic lymphohistiocytosis and/or develop into lymphoma.^[3]^ In our case, the endoscopic manifestation of multiple mass-forming lesions was exceptionally uncommon and ultimately progressed to lymphoma.

CAEAE is difficult but important to distinguish from IBD. The use of immunosuppressants and biologics in IBD often leads to opportunistic infections, including EBV infection. Using azathioprine and anti-tumor-necrosis-α antibodies increased the risk of lymphoma.^[4]^ Our patient was initially misdiagnosed with UC and underwent multiple courses of vedolizumab. Vedolizumab selectively antagonizes the α4β7 integrin, inhibiting the migration of T lymphocytes to the intestinal mucosa and alleviating inflammatory responses. This may explain the improvement in symptoms and the reduction in fecal calprotectin in this patient after vedolizumab treatment. A case-control retrospective study demonstrated that vedolizumab was associated with an increased risk of EBV reactivation in IBD.^[5]^ A study demonstrated vedolizumab did not promote EBV-driven lymphoblastoid transformation in an in vitro model of lymphomas; however, its impact in vivo remains unclear.^[6]^ Vedolizumab blocks gut lymphocyte homing of T cells, which may compromise EBV-specific T-cell immune surveillance and clearance, and could potentially contribute to the development of LPD. In this single case, whether the administration of vedolizumab combined with potential lymphocytic deficiency (history of lymphoma) played a role in the progression of CAEAE is uncertain, and the underlying mechanism remains unknown. Therefore, this is only a hypothesis that requires further investigation.

The therapeutic options for CAEAE are limited. Pharmacotherapeutic interventions demonstrate no established efficacy, such as immunomodulators, chemotherapeutic agents, and antiviral drugs.^[2]^ Patients undergoing colectomy exhibit a lower 5-year survival rate (40%, [2/5]), due to the high likelihood of surgical complications.^[7]^ At present, the only effective therapy is allo-HSCT, which eliminates lymphocytes infected or clonally proliferated by EBV through reconstituting host immunity against EBV.^[8]^ This approach can increase the long-term survival rate from approximately 25% to 55% to 65%.^[9,10]^ However, once lymphoma develops, a poor prognosis is essentially predetermined. For this patient, allo-HSCT may provide clinical benefit at the prelymphomatous stage with the lesion confined to the colon. In conclusion, it is crucial to differentiate between CAEAE and IBD. Immunosuppressants and biologics should be used with caution until a definitive diagnosis is established, as their use may accelerate the progression of CAEAE.

## Author contributions

**Formal analysis:** Tao Yang, Liang Ding, Mengjia Tao, Mengping Chen.

**Writing – original draft:** Tao Yang, Yuehua Qin.

**Writing – review & editing:** Yuehua Qin.
